# The impact of the National Heart, Lung, and Blood Institute data: analyzing published articles that used BioLINCC open access data

**DOI:** 10.12688/f1000research.21884.3

**Published:** 2021-04-21

**Authors:** Saif Aldeen AlRyalat, Osama El Khatib, Ola Al-qawasmi, Hadeel Alkasrawi, Raneem al Zu’bi, Maram Abu-Halaweh, Yara alkanash, Ibrahim Habash

**Affiliations:** 1 Department of Ophthalmology, University of Jordan Hospital, University of Jordan, Amman, 11942, Jordan; 2 Department of Internal Medicine, University of Jordan Hospital, University of Jordan, Amman, 11942, Jordan; 3 Department of Forensic Medicine, University of Jordan Hospital, The University of Jordan, Amman, 11942, Jordan

**Keywords:** Open Data, Publications, National Institute of Health, Bibliometrics

## Abstract

**Background:** Data sharing is now a mandatory prerequisite for several major funders and journals, where researchers are obligated to deposit the data resulting from their studies in an openly accessible repository. Biomedical open data are now widely available in almost all disciplines, where researchers can freely access and reuse these data in new studies. We aim to study the BioLINCC datasets, number of publications that used BioLINCC open access data, and the impact of these publications through the citations they received.

**Methods: **As of July 2019, there was a total of 194 datasets stored in BioLINCC repository and accessible through their portal. We requested the full list of publications that used these datasets from BioLINCC, and we also performed a supplementary PubMed search for other publications. We used Web of Science (WoS) to analyze the characteristics of publications and the citations they received, where WoS database index high quality articles.

**Results:** 1,086 published articles used data from BioLINCC repository for 79 (40.72%) datasets, where 115 (59.28%) datasets didn’t have any publications associated with it. Of the total publications, 987 (90.88%) articles were WoS indexed. The number of publications has steadily increased since 2002 and peaked in 2018 with a total number of 138 publications on that year. The 987 open data publications received a total of 34,181 citations up to 1 ^st^ October 2019. The average citation per item for the open data publications was 34.63. The total number of citations received by open data publications per year has increased from only 2 citations in 2002, peaking in 2018 with 2361 citations.

**Conclusion:** Majority of BioLINCC datasets were not used in secondary publications. Despite that, the datasets used for secondary publications yielded publications in WoS indexed journals and are receiving an increasing number of citations.

## Introduction

Recent years have seen an increased call for data sharing in clinical studies, especially for research funded by international and governmental agencies ^1^. The call originally aimed to maximize transparency for clinical trial results ^1^, but the benefits of data sharing extended beyond its original aim. Open access data is frequently cited as a boon for researchers, where researchers can re-analyze already collected data to answer a new research question ^2, 3^. To organize and maximize the scientific use of open access data, researchers and funders store their data in open access data repositories ^4^. The Biologic Specimen and Data Repository Information Coordinating Center (BioLINCC), is a National Heart, Lung, and Blood Institute is one such data repository, initiated in 2000 with the aim of sharing data from observational and interventional studies supported by the institute ^5^. The impact of open access data, in terms of number of datasets used from a repository, publications generated from these datasets, and citations received by these publications are still unknown. In this study, we aim to study the BioLINCC datasets, number of publications that used BioLINCC open access data, and the impact of these publications through the citations they received.

## Methods

### Data collection

**Figure 1.**
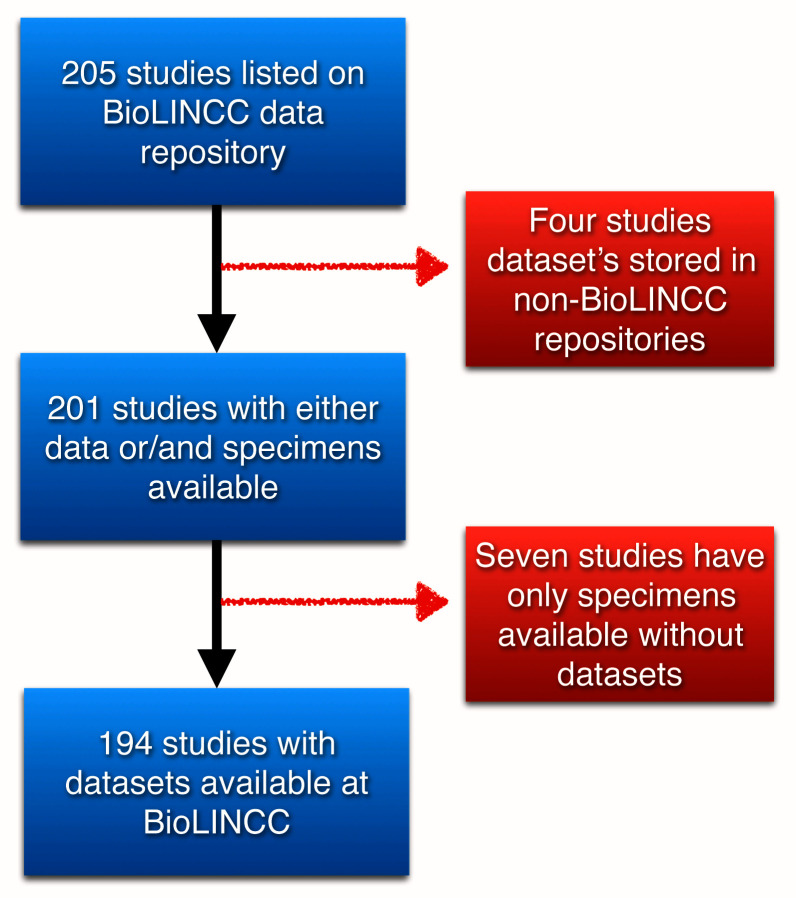
The initial datasets and the final datasets included after applying exclusion criteria.

There are a total of 205 studies listed on BioLINCC data repository, where four studies have their data stored in other repositories, and seven studies have only specimens available at the BioLINCC institution available upon request, but no datasets associated with them. We only included datasets stored in BioLINCC repository and can be accessed through their portal, which comprises 194 dataset. ( Figure 1).

We also contacted BioLINCC support to obtain an up to date list of published articles that used BioLINCC dataset, where we received a list of all publications up to 24 ^th^ July 2019. This list might not reflect the total publications of 2019, as the whole year was not included. Researchers accessing the BioLINCC datasets are requested to disclose any publication resulted from the use of the BioLINCC datasets. The BioLINCC also list published articles that used BioLINCC datasets on their website ( https://biolincc.nhlbi.nih.gov/publications/). A manual search of PubMed was also carried out to confirm an updated full list of publications, as follows:

- We used the basic search of PubMed by inputting the title of the dataset in the search field (e.g., Cooperative Study of Sickle Cell Disease or CSSCD), in order to retrieve results that mention the dataset in the title, abstract, or keywords.
- The searched articles were manually screened by one of the authors (SAA) to check if the dataset was used in the study to generate results, where authors either detail the name and acronym of dataset used in the methods section, usually with specific citation to relevant study, or in the acknowledgment section in their articles. The included articles either used data stored in the BioLINCC repository alone or used these datasets along with other datasets from other repositories
- We added the searched articles to the original dataset provided by the BioLINCC.
- We analyzed the number of studies published using each dataset (supplementary material).

### Bibliometric analysis

We used Web of Science (WoS) database to analyze the characteristics of included publications. We prepared a list of digital object identifiers (DOIs) for the included articles. We inputted the DOI list into the WoS advanced search field, where only WoS indexed publications from the total included articles were analyzed further. The WoS database has a built-in analysis to provide data regarding the number of publications using the included dataset per year (yearly publications), topic of publication, affiliation of authors, and number of citations received ^6^.

## Results

**Figure 2.**
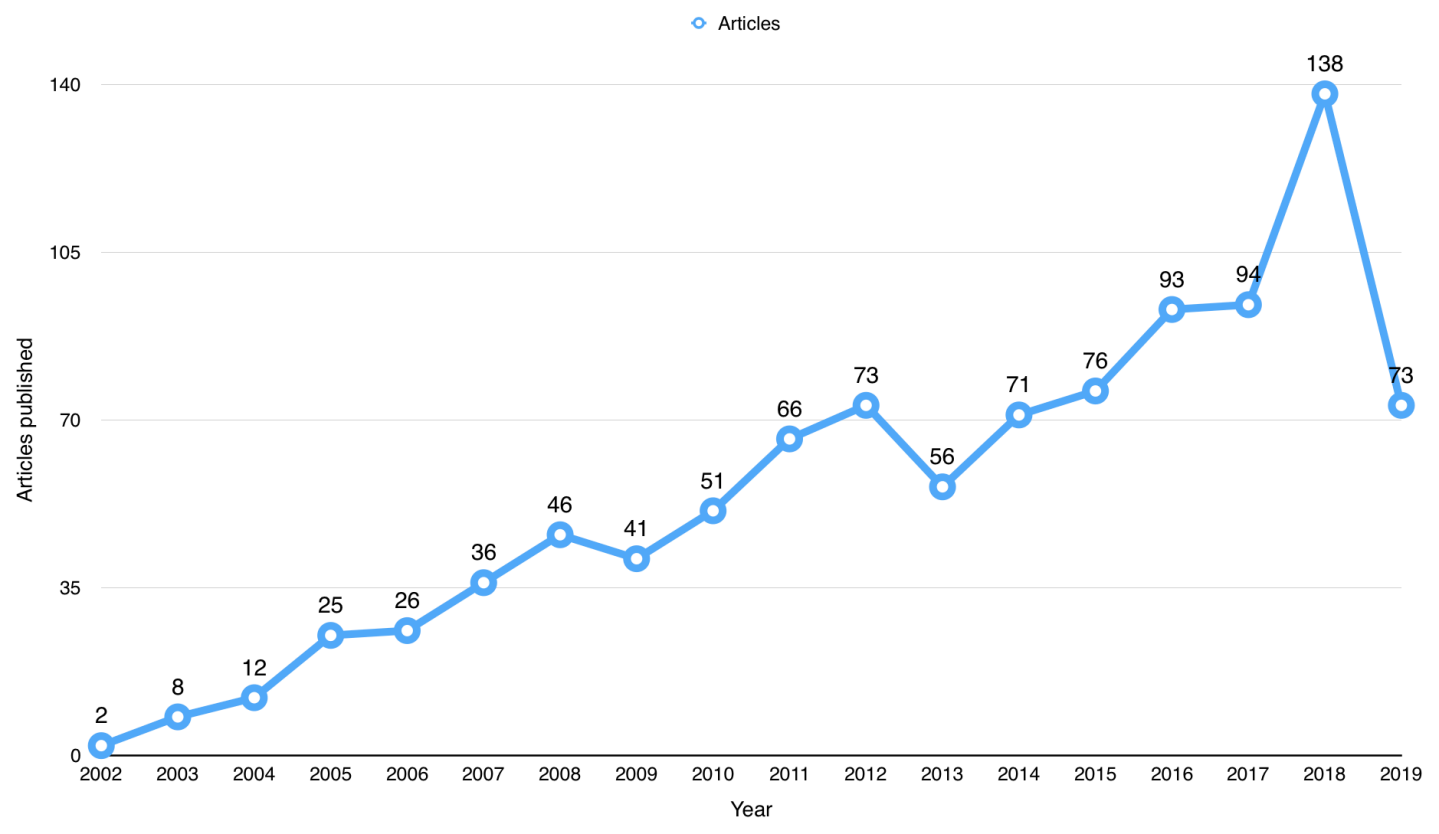
Number of publications that used Biologic Specimen and Data Repository Information Coordinating Center (BioLINCC) open data since 2002.

1,086 published articles used data from BioLINCC repository for 79 (40.72%) datasets, where 115 (59.28%) datasets didn’t have any publications associated with it. Dataset for the Atherosclerosis Risk in Communities Study (ARIC) had the highest number of publications associated with it 162 (15%), followed by Framingham Heart Study-Cohort (FHS-Cohort) with 94 (8.7%), and Cardiovascular Health Study (CHS) with 82 (7.6%). 162 (14.9%) of publications used more than one dataset ( Table 1). Out of the 1,086 published articles, only 987 (90.88%) articles were WoS indexed. All articles published were English language (see underlying data ^7^). The first publication using BioLINCC open data was from 2002. Since then, the number of publications has steadily increased since 2002, as shown in Figure 2, and peaked in 2018 with a total number of 138 publications. For the 99 (9.12%) articles that were not indexed, they were distributed over the years with the majority (i.e. 42 articles) were published in 2018.

**Table 1.** Top 10 datasets in the Biologic Specimen and Data Repository Information Coordinating Center (BioLINCC) with highest number of publications.

Dataset	Count	%
**Atherosclerosis Risk in Communities Study**	162	15.0%
**Framingham Heart Study-Cohort**	94	8.7%
**Cardiovascular Health Study (CHS)**	82	7.6%
**Digitalis Investigation Group**	76	7.0%
**Framingham Heart Study (FHS) Offspring (OS) and OMNI 1** **Cohorts**	53	4.9%
**Action to Control Cardiovascular Risk in Diabetes**	46	4.3%
**Systolic Blood Pressure Intervention Trial**	44	4.1%
**Evaluation Study of Congestive Heart Failure and** **Pulmonary Artery Catheterization Effectiveness**	39	3.6%
**Coronary Artery Risk Development in Young Adults**	38	3.5%
**Atrial Fibrillation Follow-Up Investigation of Rhythm** **Management**	33	3.1%

**Figure 3.**
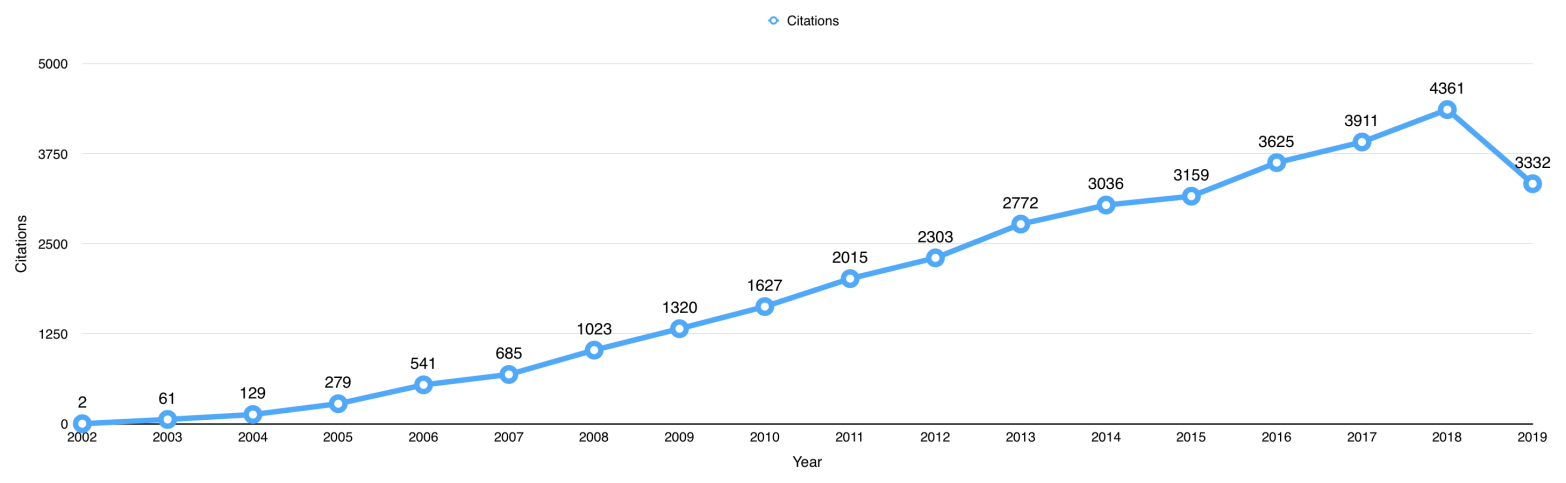
The total number of citations received by open data publications per year.

The 987 open data publications received a total of 34,181 citations from 27,904 published articles up to 1 ^st^ October 2019. The average citation per item for the publications using BioLINCC data was 34.63. The total number of citations received by publications using BioLINCC data per year has increased from only 2 citations in 2002, to a peak of 2361 citations in 2018 ( Figure 3).

**Figure 4.**
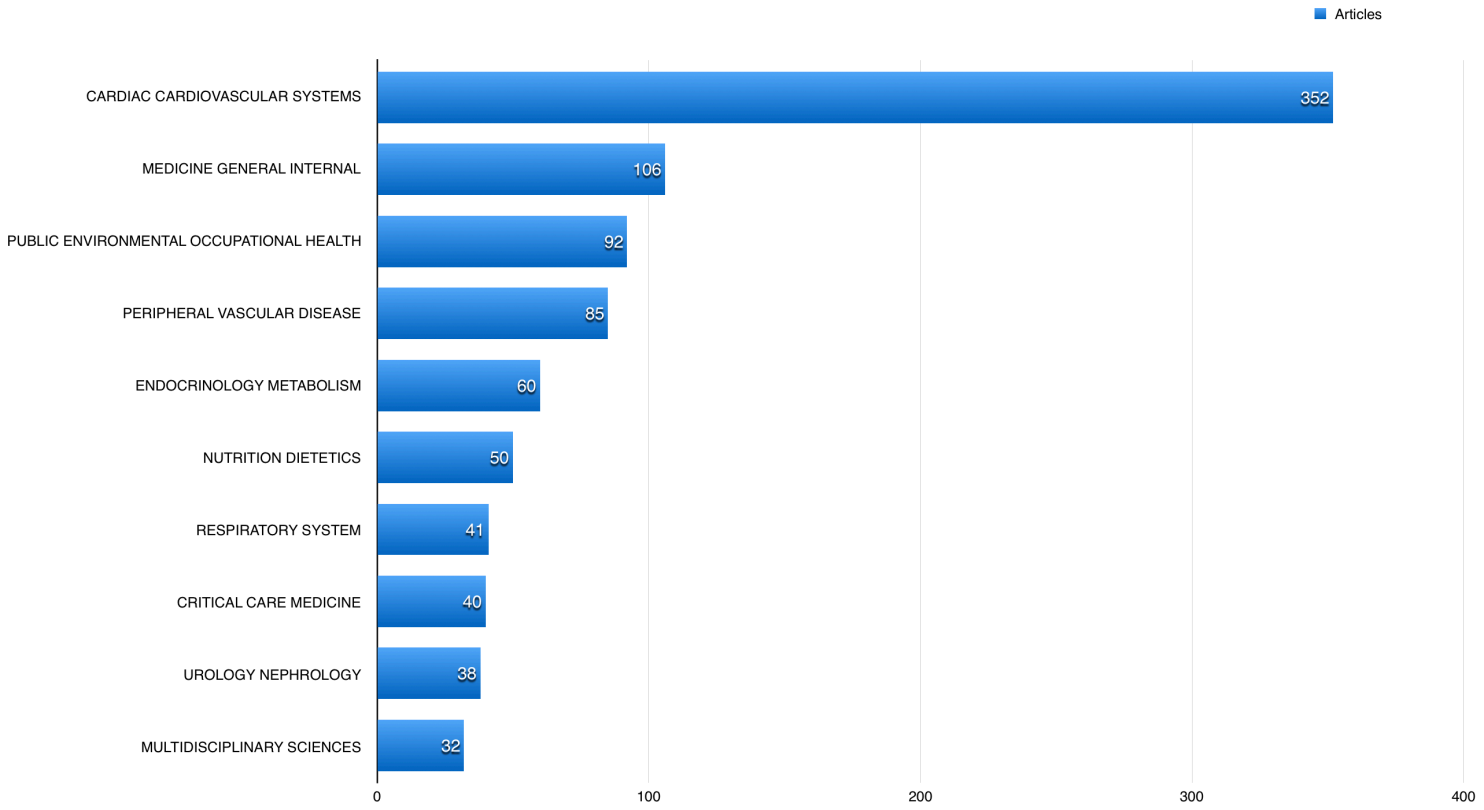
The 10 most common fields the studied open data articles published in.

**Figure 5.**
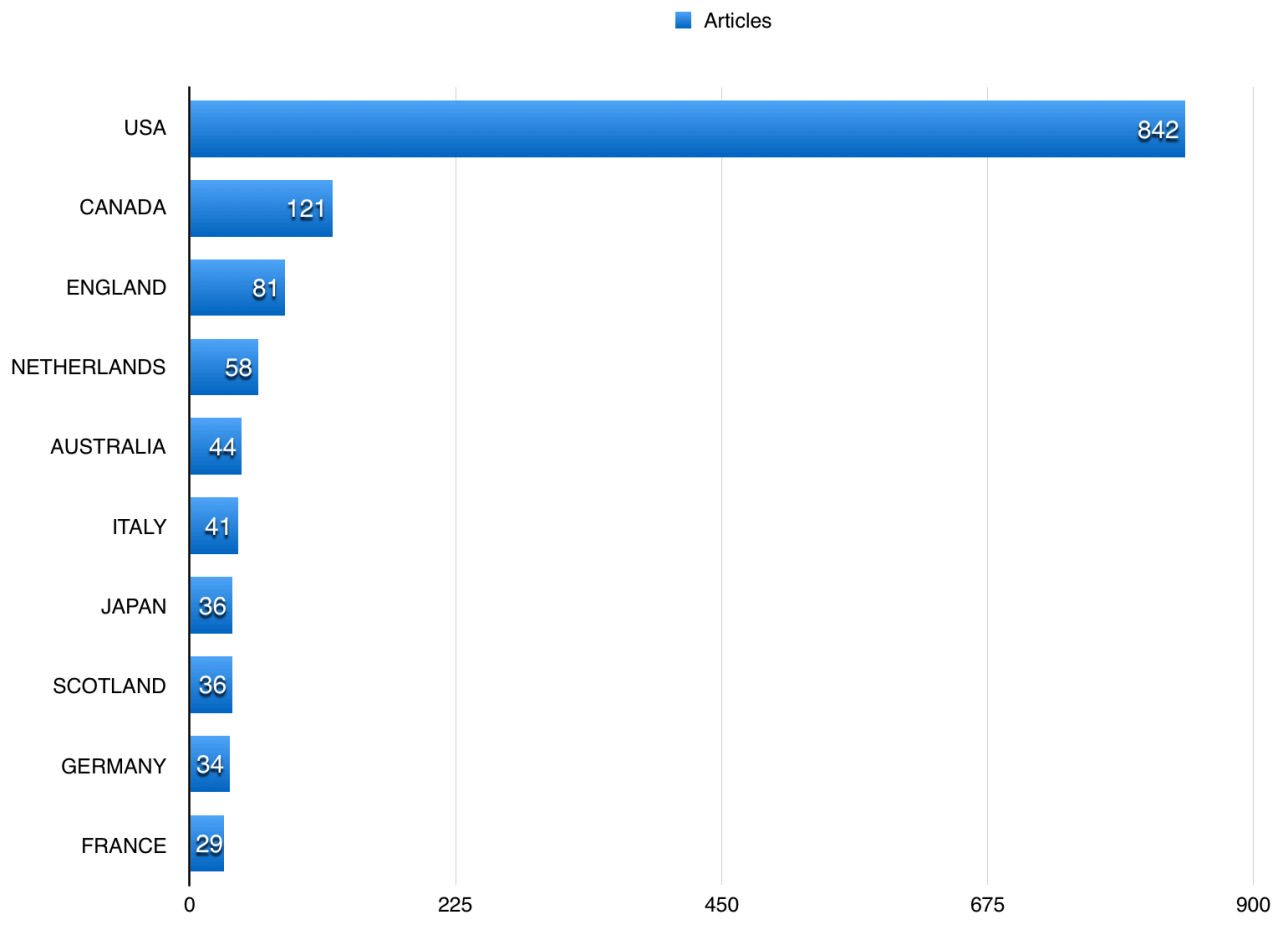
The top countries published using Biologic Specimen and Data Repository Information Coordinating Center (BioLINCC) open data.

A total of 352 (35.66%) of the published articles related to cardiac and cardiovascular systems, 106 (10.74%) articles related to general internal medicine, and 92 (9.32%) related to public and occupational health. Figure 4 shows the 10 most common fields the studied publications using BioLINCC data published in. The American Journal of Cardiology had the highest number of publications using BioLINCC data (60; 6.08%), followed by the International Journal of Cardiology with 47 (4.76%), and American Journal of Medicine 25 (2.53%). Table 2 shows the top 10 journals that publications using BioLINCC data were published in. US authors participated in 842 (85.31%) of the publications using BioLINCC data, followed by Canadian and England authors, with 121 (12.26%), and 81 (8.21%), respectively ( Figure 5). The top three affiliations in terms of publications using BioLINCC data were University of Alabama system, University of Alabama at Birmingham, and University of California system as shown in Table 3.

**Table 2.** Top 10 journals publishing articles that used Biologic Specimen and Data Repository Information Coordinating Center (BioLINCC) open data with their respective impact factor according to 2018 Journal Citation report.

JOURNAL	Impact factor	Articles (%)
**AMERICAN JOURNAL OF CARDIOLOGY**	2.843	60 (6.08%)
**INTERNATIONAL JOURNAL OF CARDIOLOGY**	3.471	47 (4.76%)
**AMERICAN JOURNAL OF MEDICINE**	4.760	25 (2.53%)
**EUROPEAN JOURNAL OF HEART FAILURE**	12.129	22 (2.23%)
**HYPERTENSION**	7.017	22 (2.23%)
**PLOS ONE**	2.776	21 (2.13%)
**CIRCULATION**	23.054	18 (1.82%)
**JOURNAL OF THE AMERICAN COLLEGE OF CARDIOLOGY**	18.639	18 (1.82%)
**JOURNAL OF CARDIAC FAILURE**	3.967	16 (1.62%)
**EUROPEAN HEART JOURNAL**	24.889	15 (1.52%)

**Table 3.** The top affiliations in terms of open data publications using Biologic Specimen and Data Repository Information Coordinating Center (BioLINCC) open data.

Organization	Articles	Percentage
**UNIVERSITY OF ALABAMA BIRMINGHAM**	240	24.316%
**UNIVERSITY OF CALIFORNIA SYSTEM**	109	11.044%
**HARVARD UNIVERSITY**	105	10.638%
**UNIVERSITY OF CALIFORNIA SAN FRANCISCO**	57	5.775%
**CASE WESTERN RESERVE UNIVERSITY**	55	5.572%
**VETERANS HEALTH ADMINISTRATION VHA**	54	5.471%
**UNIVERSITY OF CALIFORNIA LOS ANGELES**	53	5.370%
**UNIVERSITY OF TEXAS SYSTEM**	52	5.268%
**PENNSYLVANIA COMMONWEALTH SYSTEM OF HIGHER EDUCATION PCSHE**	51	5.167%

## Discussion

Tremendous effort has been made by BioLINCC in preparing dataset to be used as open data since its establishment, where hundreds of studies have been published using BioLINCC open data ^6^. Despite the finding that majority of datasets didn’t yield further publications from the re-use of the dataset, many of the datasets had high number of publications. The impact of these publications can be measured in terms of citations received, where citations of publications using BioLINCC data have dramatically increased. They received a total of 2361 citations in the year 2018. Cardiology is the main field, with more than third of publications are cardiology related, which is expected, as the dataset are related to heart, lung, blood institute. The top two journals publishing articles using BioLINCC data are also cardiology journals.

In an analysis done in 2017, Coady and his colleagues analyzed the administrative records of investigator requests for BioLINCC data, they found that 35% of clinical trial data were associated with at least one publication within five years from data public release ^8^. Our findings also showed that majority of datasets deposited in the BioLINCC repository were not associated with secondary publications. In a previous survey conducted on researchers who requested datasets from BioLINCC showed that the majority of researchers requested the data to conduct an independent research project ^8^. Moreover, Ross *et al*. in their survey also found that majority of requests to the BioLINCC repository were made by early career researchers. Where we previously pointed to the importance of open access data for underfunded and early career researchers ^2^, our results showed that the top users of open access data were from developed countries. This might be related to the fact that the data deposited and made open are from USA. Research studies performed using open access data might have important impact, an example would be the post-hoc analysis of the Digitalis Investigation Group trial using the open data of the original trial ^9^, which showed that digoxin therapy is associated with an increased risk of death from any cause among women, but not men, a finding that the original study failed to find. The digitalis trial is an example of how cardiology researchers are using open data, with efforts of cardiology initiatives encouraging data sharing and use by cardiology researchers ^10^. Clinical trial data sharing in cardiology has also been used to validate the reproducibility of published results ^11^. The high impact of publications resulted from BioLINCC shared datasets might be related to the regulations of National Institute of Health, which mandated that data collected by studies receiving more than $500,000 be stored in a publicly available repository, with BioLINCC being the main repository for NIH-NHLB institute funded research ^12^. On the other hand, data shared by platforms other than BioLINCC may lack sufficient description about the shared data, which will hamper its use by other researchers ^13^. Upon interpreting the results of the current study, several limitations need to be considered. Our results are based on BioLINCC repository, where data of well-funded research projects undergo extensive processing before being publicly shared, resulting in well-curated, high quality data. Other studies should be done to validate our results, by evaluating data repositories that do not have the pre-sharing processing. Another point here is that we used the WoS database for data extraction and analysis, which might not include several studies done using open access data from the BioLINCC repository. Moreover, we didn’t compare citations received by open data publications and primary data publications, which should be carried out in future projects. One key point that may undermine the idea of ‘impact’ of the open datasets is that the study investigators appear to be included in these counts. For example, the University of Alabama at Birmingham is a key site for some studies (e.g., CARDIA), and thus they would be publishing from their datasets whether they were open in BioLINCC or not, so this need to be considered upon interpreting the results. Finally, using citation as the sole metric for impact is a debatable issue, but it can be better used as a metric for attention.

## Data availability

### Underlying data

Harvard Dataverse: Publications that used Biologic Specimen and Data Repository Information Coordinating Center (BioLINCC) datasets. https://doi.org/10.7910/DVN/1TXA3C ^7^

This project contains the following underlying data:

- BioLINCC Dataset.tab (Spreadsheet containing details of publications using BioLINCC datasets)

Data are available under the terms of the Creative Commons Zero “No rights reserved” data waiver (CC0 1.0 Public domain dedication).
