# Supplementary material for: The National Heart, Lung, and Blood Institute data: analyzing published articles that used BioLINCC open access data
Source: F1000Res. 2021 Aug 18;9:30. Originally published 2020 Jan 20. [Version 4] doi: 10.12688/f1000research.21884.4 (PMC8447052; doi:10.12688/f1000research.21884.4)
Supplement: Peer review history

# Peer review: Reviewer response for version 4

**DOI:** 10.5256/f1000research.76584.r94247

**Published:** 2021-09-16

**Authors:** Heyam F. Dalky

**Affiliations:** 1 College of Nursing, Community and Mental Health Nursing Department, Jordan University of Science and Technology, Irbid, Jordan

This is an interesting paper about the impact of data sharing using a non-Biologic Specimen and Data Repository Information Coordinating Center (BioLINCC) .

 The authors in this manuscript analyzed previous published reports/studies that used the BioLINCC openly accessible datasets. The authors obtained their dataset mostly by asking the BioLINCC support team to provide their up to date data, and they supplemented the provided dataset by a manual search. While the manuscript was not meant to be an exhaustive study to analyze secondary publications to open data and cannot be generalized to all secondary articles published using open data, the study provided a good overview on secondary articles published using one of the highest quality open data repository.

 The authors are highly encouraged to apply a manual search conducted by the authors and needs to be further detailed, and preferably through the use of a PRISMA diagram.

 The study assessed biomedical research, so the database that more conveniently used for bibliometric analysis might be PubMed. I would advise the authors to consider PubMed database, in addition to Web of Science, in future projects concerning biomedical literature.

 As previous reviewers stated, the authors need to make sure to clarify that the study is a descriptive study and they cannot overestimate the impact of its results. The authors should work in the future on a larger project to analyze other openly accessible datasets to compare with the current results.

 The authors stated that the "Dataset for the Atherosclerosis Risk in Communities Study (ARIC) had the highest number of publications associated with it 162 (15%), followed by Framingham Heart Study-Cohort (FHS-Cohort) with 94 (8.7%), and Cardiovascular Health Study (CHS) with 82 (7.6%)." - I noticed that some major trials are deposited as multiple fragmented datasets, so it is important to consider clarifying if the authors combined such fragments when they assessed most commonly used datasets.

 The authors also stated that, "The first publication using BioLINCC open data (i.e., secondary publication) was from 2002." - it would be better to cite the publication meant by this statement.

 From the viewpoint of the reviewer, the manuscript is prepared with full attention to detail. The authors have done great efforts in presenting and comparing the data following a logical and understandable illustration. The figures enclosed make it easier for the reader to track the data and the relevant discussion.

 The work reflects highly impressed efforts in compiling data into a constructive way and presenting data in the corresponding tables. The authors complied with reviewers’ comments and considered them with attention and caution. The manuscript in its current status is highly recommended for indexing.

Is the work clearly and accurately presented and does it cite the current literature?

Yes

If applicable, is the statistical analysis and its interpretation appropriate?

Yes

Are all the source data underlying the results available to ensure full reproducibility?

Yes

Is the study design appropriate and is the work technically sound?

Yes

Are the conclusions drawn adequately supported by the results?

Yes

Are sufficient details of methods and analysis provided to allow replication by others?

Yes

Reviewer Expertise:

NA

I confirm that I have read this submission and believe that I have an appropriate level of expertise to confirm that it is of an acceptable scientific standard.

# Peer review: Reviewer response for version 4

**DOI:** 10.5256/f1000research.76584.r92162

**Published:** 2021-09-15

**Authors:** Andrew Brown, Colby Vorland

**Affiliations:** 1 Department of Applied Health Science, Indiana University School of Public Health-Bloomington, Bloomington, IN, USA

We thank the authors for revising their submission, but unfortunately concerns remain.

 The searching and screening processes are still not reproducible by readers.

- The search remains unclear. For example, if one enters their example string “Cooperative Study of Sickle Cell Disease or CSSCD” into PubMed, 141 results are found. If one enters it as ‘"Cooperative Study of Sickle Cell Disease" OR “CSSCD”’, 38 results are found.
- Over 30 papers were retrieved from the search, yet the dataset only includes 12. Which were excluded and why? For example, searching for CSSCD returns Covitz *et al.*, (1995) ^1^. The paper was not included in the supplementary file, and no reason for exclusion is given.
- No total of search results was reported, which is standard in other systematic reviews of the literature (e.g., PRISMA diagram).
- Suggesting readers should go to BioLINCC for an updated list of studies does not help the reader know which studies were available at the time of searching. The list of datasets used to produce this manuscript should be reported.

The methods for assessing which studies were classified as “open data” or “secondary publications” versus “primary data publications” are not provided. This distinction seems odd and likely undefinable for cohorts; perhaps secondary analyses of RCTs could be identified, though.

 Data formatting and documentation is still incomplete.

- It is good to see a form of data dictionary, but it includes typographical and other errors (e.g., Medical ***Subbect*** Heading), is not in any standard format, and is incomplete. Given this manuscript is on data sharing, the authors should use best practices themselves (see F.A.I.R. practices, for instance).
- Using cell formatting for additional information is bad practice. When opening the file using the previewer as a ‘.tab’ file, as referenced in the manuscript for instance, bold formatting is stripped, and that information is lost.
- Non informative missingness throughout: empty cells without justification or explanation.
- The authors mentioned a couple times that the repository will not allow for editing in their reply. If no versioning is possible, then a new repository should be made with corrected information.

There may be other concerns that we did not identify, but these were the most salient in terms of understanding what the authors did. Our general recommendation is to encourage the authors to fully and clearly disclose methods, processes, operationalization of variables, and outcomes to ensure reproducibility and transparency.

Is the work clearly and accurately presented and does it cite the current literature?

Partly

If applicable, is the statistical analysis and its interpretation appropriate?

Not applicable

Are all the source data underlying the results available to ensure full reproducibility?

Yes

Is the study design appropriate and is the work technically sound?

Partly

Are the conclusions drawn adequately supported by the results?

No

Are sufficient details of methods and analysis provided to allow replication by others?

No

Reviewer Expertise:

Meta-research

We confirm that we have read this submission and believe that we have an appropriate level of expertise to state that we do not consider it to be of an acceptable scientific standard, for reasons outlined above.

## References

[1] : The heart in sickle cell anemia. The Cooperative Study of Sickle Cell Disease (CSSCD).*Chest*.1995;108(5) : 10.1378/chest.108.5.12141214-910.1378/chest.108.5.12147587419

# Peer review: Reviewer response for version 3

**DOI:** 10.5256/f1000research.56007.r83686

**Published:** 2021-05-26

**Authors:** Andrew Brown, Colby Vorland

**Affiliations:** 1 Department of Applied Health Science, Indiana University School of Public Health-Bloomington, Bloomington, IN, USA

We thank the authors for responding to our comments. We still have several outstanding concerns.

 Specifically, we thank the authors for clarifying about the 9% of articles not indexed in WoS. Publishing the full results of the distribution of WoS indexing over time (even if like in Figure 2) would aid interpretation. Given that 2018 was the last full year included, it seems to support the point that your analysis is underestimating papers using BioLINCC in recent years because there may be a delay in WoS indexing. This should be listed as a limitation in the discussion section.

 The description of methods is improved, although still not reproducible. On what day was the search performed? It is not clear from the authors’ published dataset what the BioLINCC dataset titles are or how they determined exact search strings. For instance, did all datasets have acronyms like the example used in the authors’ reply to our review (Cooperative Study of Sickle Cell Disease or CSSCD)? How many total results were returned and how many articles were screened manually?

 We re-emphasize that conclusions about the “impact” of BioLINCC data are not appropriate. It is possible that, relatively speaking, there has been no increase in the use of BioLINCC data relative to using other repositories, or compared to authors using their own datasets. Metrics of use may easily just reflect increasing trends of total publications over time. Just because a dataset is included in BioLINCC is not an indication of an impact of BioLINCC. It is reasonable that a central repository would facilitate data sharing and use, but this has not been shown in this descriptive analysis. Without an appropriate comparator group, the results are descriptive, and the interpretation is limited to descriptions, not of impact.

 Regarding the data:

- The dataset still lacks a codebook as far as we could find. A codebook includes descriptions of each variable name in the file so others can interpret what each column is (for example, what is ‘recid’…).
- It is still not clear where rows without DOIs came from. They were papers provided by the BioLINCC team that did not contain DOIs and so they were not retrieved from WoS?
- There are rows inexplicably bolded.
- Column called ‘url’ appears to have WoS syntax.
- The ‘studylist’ column is not machine readable and unclearly delimited (e.g., are datasets comma-separated? Are datasets counted separately for studies like WHI-CT and WHI-OS?).
- Missing data are not explained.
- The entire list of datasets from BioLINCC are missing, and thus the ones without publications cannot be confirmed. The authors say that the majority of datasets do not have a publication associated, which seems unlikely.

The authors state: “Other studies should be done to validate our results, by evaluating data repositories that do not have the pre-sharing processing.” The authors only looked at and discuss BioLINCC, without much generalization (or generalizability), so it is unclear what conclusions would be ‘validated’.

 What do the authors mean by “open data publications and primary data publications”?

 Regarding Alabama: please confirm whether it should be the University of Alabama System or University of Alabama at Birmingham in the figure and text.

 We note the authors included a sentence directly from our review: “For example, the University of Alabama at Birmingham is a key site for some studies (e.g., CARDIA), and thus they would be publishing from their datasets whether they were open in BioLINCC or not”. While we are glad the authors took our concerns to heart, we are not sure what to think about our sentence being lifted directly.

 Number of citations in text (2361) does not match figure for 2018.

 Some grammatical concerns: Figure 1: Should say “Four studies’ datasets”; in the text should be “comprises 194 datasets” instead of dataset; elsewhere “that used BioLINCC dataset” should be “datasets”. “they were distributed over the years with the majority (i.e. 42 articles) were published in 2018” should not have the second “were”. “English authors” not “England authors”. Acronyms for NIH/NHLBI never established but used in discussion. Formal English avoids contractions (e.g., “didn’t”); and so forth.

Is the work clearly and accurately presented and does it cite the current literature?

Partly

If applicable, is the statistical analysis and its interpretation appropriate?

Not applicable

Are all the source data underlying the results available to ensure full reproducibility?

Yes

Is the study design appropriate and is the work technically sound?

Partly

Are the conclusions drawn adequately supported by the results?

No

Are sufficient details of methods and analysis provided to allow replication by others?

No

Reviewer Expertise:

Meta-research

We confirm that we have read this submission and believe that we have an appropriate level of expertise to state that we do not consider it to be of an acceptable scientific standard, for reasons outlined above.

# Response

**Published:** 2021-08-13

**Authors:** Saif Aldeen AlRyalat

**Affiliations:** University of Jordan, Jordan

The reviewers again performed an in-depth assessment and provided valuable points to be amended and improved, which we followed point by point. They also suggested improvements in the dataset. In this regard, we uploaded a separate codebook to detail the dataset details. We hope the manuscript in its current improved version satisfies, to a certain degree, their expectations.

*We thank the authors for responding to our comments. We still have several outstanding concerns.*

*Specifically, we thank the authors for clarifying about the 9% of articles not indexed in WoS. Publishing the full results of the distribution of WoS indexing over time (even if like in Figure 2) would aid interpretation. Given that 2018 was the last full year included, it seems to support the point that your analysis is underestimating papers using BioLINCC in recent years because there may be a delay in WoS indexing. This should be listed as a limitation in the discussion section.*

**Reply:** We agree with the reviewers that delayed indexing by WoS might lead to underestimation in the number of WoS indexed articles. We further clarified this in the article’s limitations.

*The description of methods is improved, although still not reproducible. On what day was the search performed? It is not clear from the authors’ published dataset what the BioLINCC dataset titles are or how they determined exact search strings. For instance, did all datasets have acronyms like the example used in the authors’ reply to our review (Cooperative Study of Sickle Cell Disease or CSSCD)? How many total results were returned and how many articles were screened manually?*

**Reply:** Thank you for the suggestions that led to this improvement in your previous revision. The PubMed search was carried out in the next day directly (i.e., on 25 ^th^ of July 2019), and the search results were saved and screened during subsequent days. All datasets had acronyms as shown in the column (studylist), and as evident on the BioLINCC own website.

 In regard to exact numbers, we did not record them at the time of search, so they are not available for reporting. We clarified these points in the methods.

*We re-emphasize that conclusions about the “impact” of BioLINCC data are not appropriate. It is possible that, relatively speaking, there has been no increase in the use of BioLINCC data relative to using other repositories, or compared to authors using their own datasets. Metrics of use may easily just reflect increasing trends of total publications over time. Just because a dataset is included in BioLINCC is not an indication of an impact of BioLINCC. It is reasonable that a central repository would facilitate data sharing and use, but this has not been shown in this descriptive analysis. Without an appropriate comparator group, the results are descriptive, and the interpretation is limited to descriptions, not of impact.*

**Reply:** We agree with the reviewer on the importance of not overestimating the results of our study. In the previous revision, we tried to emphasize on this point in the limitations. Now, we further reviewed the study to explicitly replace words like “impact” by other appropriate words that reflect the descriptive nature of this study, including the word “impact” in the title.

*Regarding the data:*

- *The dataset still lacks a codebook as far as we could find. A codebook includes descriptions of each variable name in the file so others can interpret what each column is (for example, what is ‘recid’…).*

**Reply:** The details about what each column title reflect were already provided in the “Notes” section on dataverse website. However, we agree with the reviewer that a more explicite and detailed codebook still needed, so we uploaded a one that can be downloaded at the dataverse website.

- *It is still not clear where rows without DOIs came from. They were papers provided by the BioLINCC team that did not contain DOIs and so they were not retrieved from WoS?*

**Reply:** They are provided by the BioLINCC but with missing doi as the reviewers stated, but we filled the doi during the WoS search, so they were retrieved from WoS. The point here is that we did not add the doi to the original dataset.

- *There are rows inexplicably bolded.*

**Reply: **Bolded rows are for non-journal publications, including thesis and book chapters. We bolded them so that researchers can easily identify them and exclude them if their research was on original articles only. We included such explanation in the codebook.

- *Column called ‘url’ appears to have WoS syntax.*

**Reply:** url provide a direct link to the publication, or a web of science number to access the publication through. We detailed this in the codebook. The repository prohibit any new edits on the uploaded dataset due to our previous amendments.

- *The ‘studylist’ column is not machine readable and unclearly delimited (e.g., are datasets comma-separated? Are datasets counted separately for studies like WHI-CT and WHI-OS?).*

**Reply:** The studylist column contain acronyms for datasets used in the publications. They are listed in a csv format as the reviewers correctly stated. They can be separated in different columns if a researcher wishes to analyze them specifically.

- *Missing data are not explained.*

**Reply:** Upon inputting data in a bibliometric database like WoS or PubMed, data will be automatically retrieved. Doi and PMID are the important fields to retrieve such data. Missing doi and PMID can be recovered using publication’s title. Due to the restrictions by the data repository we deposited in, we are unable to edit the dataset uploaded.

- *The entire list of datasets from BioLINCC are missing, and thus the ones without publications cannot be confirmed. The authors say that the majority of datasets do not have a publication associated, which seems unlikely.*

**Reply:** The study was mainly based on data provided by BioLINCC support team, supplemented by our manual search. BioLINCC mandate yearly report along with immediate reporting of any publication resulted from their datasets, which makes their input a reliable one. The fill BioLINCC dataset list is available at their website.

*The authors state: “Other studies should be done to validate our results, by evaluating data repositories that do not have the pre-sharing processing.” The authors only looked at and discuss BioLINCC, without much generalization (or generalizability), so it is unclear what conclusions would be ‘validated’.*

**Reply:** The main point here is for readers to keep in mind that our descriptive study is focused on BioLINCC data repository, and might not reflect other data repositories. We re-phrased the sentence to be “Other studies should be done to evaluate data repositories that do not have the pre-sharing processing.”

*What do the authors mean by “open data publications and primary data publications”?*

**Reply:** Open data publications are also known as secondary publications that used an openly accessible dataset, while primary data publications are those that were published using the original data collected for their purpose. We clarified this in the text.

*Regarding Alabama: please confirm whether it should be the University of Alabama System or University of Alabama at Birmingham in the figure and text.*

**Reply:** It was University of Alabama at Birmingham. Clarified in the text.

*We note the authors included a sentence directly from our review: “For example, the University of Alabama at Birmingham is a key site for some studies (e.g., CARDIA), and thus they would be publishing from their datasets whether they were open in BioLINCC or not”. While we are glad the authors took our concerns to heart, we are not sure what to think about our sentence being lifted directly.*

**Reply:** This is an opportunity to thank the reviewers for the sentence that fitted in its context. The reviewers put an extensive effort in this review, and such sentence was “perfect” for the context.

*Number of citations in text (2361) does not match figure for 2018.*

**Reply:** We corrected the mistake.

*Some grammatical concerns: Figure 1: Should say “Four studies’ datasets”; in the text should be “comprises 194 datasets” instead of dataset; elsewhere “that used BioLINCC dataset” should be “datasets”. “they were distributed over the years with the majority (i.e. 42 articles) were published in 2018” should not have the second “were”. “English authors” not “England authors”. Acronyms for NIH/NHLBI never established but used in discussion. Formal English avoids contractions (e.g., “didn’t”); and so forth.*

**Reply:** Thank you for the suggestions. We corrected the pointed mistakes, as suggested,

# Peer review: Reviewer response for version 2

**DOI:** 10.5256/f1000research.29748.r72105

**Published:** 2020-10-19

**Authors:** Lisa Federer

**Affiliations:** 1 National Library of Medicine, National Institutes of Health, Bethesda, MD, USA

I appreciate the authors' revisions.

Is the work clearly and accurately presented and does it cite the current literature?

Partly

If applicable, is the statistical analysis and its interpretation appropriate?

Not applicable

Are all the source data underlying the results available to ensure full reproducibility?

Yes

Is the study design appropriate and is the work technically sound?

Partly

Are the conclusions drawn adequately supported by the results?

Yes

Are sufficient details of methods and analysis provided to allow replication by others?

Yes

Reviewer Expertise:

data science, data sharing and reuse

I confirm that I have read this submission and believe that I have an appropriate level of expertise to confirm that it is of an acceptable scientific standard.

# Peer review: Reviewer response for version 1

**DOI:** 10.5256/f1000research.24126.r70340

**Published:** 2020-09-25

**Authors:** Andrew Brown, Colby Vorland

**Affiliations:** 1 Department of Applied Health Science, Indiana University School of Public Health-Bloomington, Bloomington, IN, USA

**Summary:**

 The authors ask an interesting question as to what the impact of BioLINCC has been on the use of open data. However, the assessments of impact do not seem to appropriately contextualize the use of BioLINCC datasets as compared to growth of scientific publishing overall. Further, the authors include data in their analyses before the existence of BioLINCC, and the methods used to sample are unclear.

**--------------**

**Abstract**

 It is unclear why WoS indexing is in the conclusions, unless it is being used as a proxy for ‘impact.’ If so, the article does not make clear that WoS indexing is being used as a sign of impact.

 The choice of ‘citations’ as a metric for ‘impact’ is questionable. Citations may best be considered a metric of ‘attention’.

**Introduction**

 BioLINCC was not initiated in 2000 as stated- it was 2008: https://www.nhlbi.nih.gov/science/biologic-specimen-and-data-repository-information-coordinating-center-biolincc

 The NHLBI had a different repository since 2000. However, since the authors focus on BioLINCC, this raises the question why the authors start their survey in 2002, and how this data came to be included in their sampling.

**Methods**

 The methods as currently stated are not reproducible. For example, on what date were BioLINCC and PubMed sampled? What was the search string for PubMed?

 It is not clear whether PubMed entries necessarily indicate that they use BioLINCC datasets. The authors mention “any study that reported the use of the searched dataset as part of its results was included”. Does that mean full texts were reviewed? If so, by what process and by which of the authors?

 It is not clear what is meant by “title of the dataset in the search field”. Does this mean the study name? The study acronym? It seems likely that many publications would not use the exact dataset name in the title or abstract and this approach would therefore potentially miss papers. Were any new papers found beyond the BioLINCC list from the PubMed search? How many articles from the BioLINCC list were not confirmed in the PubMed search? This information is missing from the methods.

**Results**

 It is indicated that over 9% of the articles using data from BioLINCC are not WoS indexed. If these articles are not evenly distributed over time, then it will skew the results of the trends. For example – were the papers not indexed more recent papers that WoS has not yet picked up? At minimum, the authors can manually extract the year, journal, and country of publication from these papers to include the in assessments.

 The utility of the analyses as currently presented seem questionable. How does the increase in articles published and citations by year compare to trends in overall metrics of these measures? i.e. do these trends outpace or just reflect the growth of scientific publishing overall? The authors may also consider limiting such comparison to the specific fields that use BioLINCC data.

 The authors note the values ‘peaked in 2018’, but that was the most recent year of full data, given their partial year in 2019. Thus, 2019 is likely artificially small by virtue of it being a partial year.

 The University of Alabama at Birmingham is part of the University of Alabama System, and thus counting them separately does not seem to make sense.

 It is unclear how fields of study were determined. Were these just extracted from WoS (is this “topic of publication” per methods or a separate extraction), or did the authors classify them? Regardless, the finding that cardiology is the top field is not surprising, given that BioLINCC is from the National Heart, Lung, and Blood Institute. This should be made clear.

 One key point that may undermine the idea of ‘impact’ of the open datasets is that the study investigators appear to be included in these counts. For example, the University of Alabama at Birmingham is a key site for some studies (e.g., CARDIA), and thus they would be publishing from their datasets whether they were open in BioLINCC or not. So, what is the incremental contribution to investigators who are not part of the cohort? What difference is it making for how many papers would be published if the data were open or not?

**Discussion**

 In general, the discussion does not seem to flow logically. For example, in one paragraph, the authors discuss the percent of publications after data release, the top countries from which BioLINCC data are used and top journals, and then a single example of clinical impact from using BioLINCC data. The points in the discussion should be separated and connected to the purpose of the study. New results (e.g., impact factor) should not be introduced in the discussion. Further, have there been other studies that have examined these or related questions about BioLINCC or other repositories?

 “The impact of these publications can be measured in terms of citations received, where citations of publications using BioLINCC data have exponentially increased”

- Exponential growth is a specific mathematical term whereas the growth in the figures appears to be roughly linear.

“Researchers new to open data might be skeptical about the publishing opportunity of studies performed using open data.”

- This statement does not seem relevant to the analysis nor supported by any citations.

Finally, a limitations section is needed noting the sole focus on WoS and whether the inclusion of other indexes might alter conclusions. For example – to our knowledge, F1000Research is not indexed in WoS; would relevant studies published here be included in a different index?

**Data**

 We downloaded and inspected the data:

- There is no data dictionary to interpret the dataset.
- ‘Recid’ starts at 4 and not 1. Some ‘Recid’s are missing (for example, #5, #7). Were these entries those that were not indexed by WoS? Those DOIs would still be useful to include in the dataset so future researchers can use them.
- Were theses and other article types included in all analyses (include this information in the methods)?
- There are missing data (e.g., funding; MESH terms; article types; study type; one publication was missing ‘study list’).
- The authors state that they searched WoS by DOI, and yet DOIs are missing from some entries. How was this accounted for in the analysis? Are the missing DOIs counted as part of ‘not indexed in WoS’?

**General**

 The writing is generally clear, but it could benefit from a grammatical edit in some passages.

Is the work clearly and accurately presented and does it cite the current literature?

Partly

If applicable, is the statistical analysis and its interpretation appropriate?

Not applicable

Are all the source data underlying the results available to ensure full reproducibility?

Yes

Is the study design appropriate and is the work technically sound?

Partly

Are the conclusions drawn adequately supported by the results?

No

Are sufficient details of methods and analysis provided to allow replication by others?

No

Reviewer Expertise:

Meta-research

We confirm that we have read this submission and believe that we have an appropriate level of expertise to state that we do not consider it to be of an acceptable scientific standard, for reasons outlined above.

# Response

**Published:** 2021-04-08

**Authors:** Saif Aldeen AlRyalat

**Affiliations:** University of Jordan, Jordan

I went through the manuscript and amended and responded to all comments. Here are the responses.

**Reviewer** **Colby Vorland and** **Andrew Brown**

 It is an honor to receive a feedback from Drs Colby and Brown from Indiana university, we performed almost all the changes suggested, and we hope the current version satisfy the quality required. Here are the detailed responses.

 Summary:

 The authors ask an interesting question as to what the impact of BioLINCC has been on the use of open data. However, the assessments of impact do not seem to appropriately contextualize the use of BioLINCC datasets as compared to growth of scientific publishing overall. Further, the authors include data in their analyses before the existence of BioLINCC, and the methods used to sample are unclear.

**Response**: Thank you. The study is mostly descriptive of the studies published using datasets stored at the BioLINCC repository, with a bibliometric analysis of these studies. We believe that such analysis will show the impact of open data and will encourage authors to further share their data publicly. So we agree with the reviewer that the current analysis lacks the comparison with the growth of overall scientific literature, but we will consider such analysis in the near future.

 The BioLINCC is basically a repository to store and facilitate the share of data collected by National Heart, Lung, and Blood Institute funded studies, where these studies and their data might have been done before the existence of the BioLINCC, but were stored in the BioLINCC repository afterward.

**--------------**

**Abstract**

 It is unclear why WoS indexing is in the conclusions, unless it is being used as a proxy for ‘impact.’ If so, the article does not make clear that WoS indexing is being used as a sign of impact.

**Response**: We used the WoS database as they have strict and high bar criteria to index publication, so it was used as a proxy for impact as the authors stated. We clarified this point in the abstract as suggested.

 The choice of ‘citations’ as a metric for ‘impact’ is questionable. Citations may best be considered a metric of ‘attention’.

**Response**: we agree with the author that the issue of considering citation as the sole metric for impact is debatable, so we made this point clear in the limitation section.

 Introduction

 BioLINCC was not initiated in 2000 as stated- it was 2008: https://www.nhlbi.nih.gov/science/biologic-specimen-and-data-repository-information-coordinating-center-biolincc

 The NHLBI had a different repository since 2000. However, since the authors focus on BioLINCC, this raises the question why the authors start their survey in 2002, and how this data came to be included in their sampling.

**Response**: Whereas the BioLINCC repository itself was made in 2008, the datasets deposited in it were developed before that, since 2000*, so the publications may date back to as early as 2002.

 * Coady SA, Wagner E. Sharing individual level data from observational studies and clinical trials: a perspective from NHLBI. Trials. 2013 Dec;14(1):1-3.

 Methods

 The methods as currently stated are not reproducible. For example, on what date were BioLINCC and PubMed sampled? What was the search string for PubMed?

 It is not clear whether PubMed entries necessarily indicate that they use BioLINCC datasets. The authors mention “any study that reported the use of the searched dataset as part of its results was included”. Does that mean full texts were reviewed? If so, by what process and by which of the authors?

**Response**: The original dataset was extracted by contacting the BioLINCC personnel and asking them for the up to date list of publications that used repository’s datasets. Our supplementary PubMed search was carried out as follows, which we further elaborated in the methods section:

 “A manual search of PubMed was also carried out to confirm an updated full list of publications as follows:

- We used the basic search of PubMed by inputting the title of the dataset in the search field (e.g., Cooperative Study of Sickle Cell Disease or CSSCD), in order to retrieve results that mention the dataset in the title, abstract, or keywords.
- The searched articles were manually screened by one of the authors (SAA) to check if the dataset was used in the study to generate results, where authors either detail the name and acronym of dataset used in the methods section, usually with specific citation to relevant study, or in the acknowledgment section in their articles. The included articles either used data stored in the BioLINCC repository alone or used these datasets along with other datasets from other repositories
- We added the searched articles to the original dataset provided by the BioLINCC.
-  We analyzed the number of studies published using each dataset (supplementary material).”

It is not clear what is meant by “title of the dataset in the search field”. Does this mean the study name? The study acronym? It seems likely that many publications would not use the exact dataset name in the title or abstract and this approach would therefore potentially miss papers. Were any new papers found beyond the BioLINCC list from the PubMed search? How many articles from the BioLINCC list were not confirmed in the PubMed search? This information is missing from the methods.

**Response**: Inputting the title and the acronym of the dataset in the PubMed search will retrieve all articles that mentioned the dataset in the title, abstract, keywords. The guidelines for reporting secondary analysis articles require the mention of the dataset used in the title or abstract*. Despite that, we agree with the reviewers that our search might miss few articles that did not mention the dataset there. We tried to limit the words for the methods and results, which is why these details are not provided the full manuscript. We directly added the results searched by the supplementary search directly on the original dataset provided by the BioLINCC, which is provided as supplementary material.

 * Swart E, Schmitt J. STandardized Reporting Of Secondary data Analyses (STROSA)-Vorschlag für ein Berichtsformat für Sekundärdatenanalysen. Zeitschrift für Evidenz, Fortbildung und Qualität im Gesundheitswesen. 2014 Jan 1;108(8-9):511-6.

 Results

 It is indicated that over 9% of the articles using data from BioLINCC are not WoS indexed. If these articles are not evenly distributed over time, then it will skew the results of the trends. For example – were the papers not indexed more recent papers that WoS has not yet picked up? At minimum, the authors can manually extract the year, journal, and country of publication from these papers to include the in assessments.

**Response**: We analyzed the non-indexed articles manually to check if they were published in 2019, which if so might reflect a delay in the indexing. We found that they were distributed over the years with the majority were published in the year 2018. We could not perform detailed analysis as they could not be analyzed using the WoS database, so clarified this in the results: “For the 99 (9.12%) articles that were not indexed, they were distributed over the years with the majority (i.e. 42 articles) were published in 2018.”

 The utility of the analyses as currently presented seem questionable. How does the increase in articles published and citations by year compare to trends in overall metrics of these measures? i.e. do these trends outpace or just reflect the growth of scientific publishing overall? The authors may also consider limiting such comparison to the specific fields that use BioLINCC data.

**Response**: Thank you for the important point. While we did not compare with the overall publishing trend in the field, we tried to show the increase in the number of publication using open access data in each field. The use of specific dataset might not be restricted to the field of the dataset itself, as a dataset that was originally a cardiovascular dataset might be used by researchers from other fields for other ideas. As an example, Radiological images in ACCESS datasets were used several times for Radiology publications.

 The authors note the values ‘peaked in 2018’, but that was the most recent year of full data, given their partial year in 2019. Thus, 2019 is likely artificially small by virtue of it being a partial year.

**Response**: We agree with the reviewer, so we made this point clear in the methods. 

 The University of Alabama at Birmingham is part of the University of Alabama System, and thus counting them separately does not seem to make sense.

**Response**: We corrected according to reviewer suggestion, the WoS database have both as separate affiliation, which led to this confusion.

 It is unclear how fields of study were determined. Were these just extracted from WoS (is this “topic of publication” per methods or a separate extraction), or did the authors classify them? Regardless, the finding that cardiology is the top field is not surprising, given that BioLINCC is from the National Heart, Lung, and Blood Institute. This should be made clear.

**Response**: These are WoS based classification, we changed in the text accordingly.

 One key point that may undermine the idea of ‘impact’ of the open datasets is that the study investigators appear to be included in these counts. For example, the University of Alabama at Birmingham is a key site for some studies (e.g., CARDIA), and thus they would be publishing from their datasets whether they were open in BioLINCC or not. So, what is the incremental contribution to investigators who are not part of the cohort? What difference is it making for how many papers would be published if the data were open or not?

**Response**: We thank the reviewers for the important remarks, as it is difficult to performed such discrimination in the current study, we made this point clear in the limitation part, so that readers would consider this point upon interpreting the results.

 Discussion

 In general, the discussion does not seem to flow logically. For example, in one paragraph, the authors discuss the percent of publications after data release, the top countries from which BioLINCC data are used and top journals, and then a single example of clinical impact from using BioLINCC data. The points in the discussion should be separated and connected to the purpose of the study. New results (e.g., impact factor) should not be introduced in the discussion. Further, have there been other studies that have examined these or related questions about BioLINCC or other repositories?

**Response**: We made several changes on the discussion to improve its flow. We removed some of the unrelated discussion part. We also removed the part related to impact factor.

 “The impact of these publications can be measured in terms of citations received, where citations of publications using BioLINCC data have exponentially increased”

 Exponential growth is a specific mathematical term whereas the growth in the figures appears to be roughly linear.

**Response**: We changed accordingly, thank you.

 “Researchers new to open data might be skeptical about the publishing opportunity of studies performed using open data.”

 This statement does not seem relevant to the analysis nor supported by any citations.

**Response**: We removed it through our effort to improve the discussion part, thank you.

 Finally, a limitations section is needed noting the sole focus on WoS and whether the inclusion of other indexes might alter conclusions. For example – to our knowledge, F1000Research is not indexed in WoS; would relevant studies published here be included in a different index?

**Response**: We agree with the reviewer, we made clear that WoS database might not include all studies published using open access data from BioLINCC repository.

 Data

 We downloaded and inspected the data:

 There is no data dictionary to interpret the dataset.

**Response**: We added a description at the dataset website:

https://dataverse.harvard.edu/dataset.xhtml?persistentId=doi%3A10.7910%2FDVN%2F1TXA3C&version=DRAFT

 ‘Recid’ starts at 4 and not 1. Some ‘Recid’s are missing (for example, #5, #7). Were these entries those that were not indexed by WoS? Those DOIs would still be useful to include in the dataset so future researchers can use them.

**Response**: The entries did not use the BioLINCC data, so were not included in the dataset.

 Were theses and other article types included in all analyses (include this information in the methods)?

**Response**: Thesis were not included, we added this to the methods. 

 There are missing data (e.g., funding; MESH terms; article types; study type; one publication was missing ‘study list’). The authors state that they searched WoS by DOI, and yet DOIs are missing from some entries. How was this accounted for in the analysis? Are the missing DOIs counted as part of ‘not indexed in WoS’?

**Response**: Missing doi were added to the manually to the WoS search for data analysis. They were not counted as part of the “not indexed in WoS”. After inputting doi to the databse, information about the study will automatically be retrieved from the WoS database, so missing data in the excel sheet won’t affect the analyzed data.

 Thank you for your consideration!

 Sincerely,

 Saif Aldeen AlRyalat, M.D.

 Corresponding author.

# Peer review: Reviewer response for version 1

**DOI:** 10.5256/f1000research.24126.r70339

**Published:** 2020-09-08

**Authors:** Lisa Federer

**Affiliations:** 1 National Library of Medicine, National Institutes of Health, Bethesda, MD, USA

The authors have addressed an interesting question - what are the impacts of open data, specifically considering citations received by publications using open data sets. This question is very timely given the increasing number of funder and journal requirements that datasets be made open. However, my primary concern with this paper is that it doesn't really provide much context for understanding impact. 

 The authors have tracked citations to articles that reuse datasets over time. However, with nothing to compare these counts to, it's hard to contextualize what these citations really mean. Are these papers being cited more/less than similar articles that aren't reusing datasets? How do citations to these articles describing secondary reuse compare to the number of citations received by the articles describing the dataset originally and its primary use? It's evident that citations to these articles are going up over time, but that's to be expected to an extent. So I'm not really sure what to make of these numbers and how to use them to understand impact. While this article provides an overview of the state of citations to articles reusing BioLINCC data, it is unclear to me what conclusions can be reasonably drawn from this analysis about impact.

Is the work clearly and accurately presented and does it cite the current literature?

Partly

If applicable, is the statistical analysis and its interpretation appropriate?

Not applicable

Are all the source data underlying the results available to ensure full reproducibility?

Yes

Is the study design appropriate and is the work technically sound?

Partly

Are the conclusions drawn adequately supported by the results?

Yes

Are sufficient details of methods and analysis provided to allow replication by others?

Yes

Reviewer Expertise:

data science, data sharing and reuse

I confirm that I have read this submission and believe that I have an appropriate level of expertise to state that I do not consider it to be of an acceptable scientific standard, for reasons outlined above.

# Response

**Published:** 2020-09-18

**Authors:** Saif Aldeen AlRyalat

**Affiliations:** University of Jordan, Jordan

We would like to thank Dr. Frederer, who is an expert in the field of data science, for the insight and thoughts she shared through her revision. While we agree with her comments on the gap between our study and the big aim of “studying the impact of open data”. Our study tried to answer a certain aspect of this aim, which we further specified in the current version, and added more data that will provide better insight on BioLINCC datasets, publications, and their impact. Here are our detailed responses:

**Comment**: The authors have addressed an interesting question - what are the impacts of open data, specifically considering citations received by publications using open data sets. This question is very timely given the increasing number of funder and journal requirements that datasets be made open. However, my primary concern with this paper is that it doesn't really provide much context for understanding impact. 

**Response**: We totally agree with Dr. Federer, that the analysis of open data is most relevant during this time. One of the most important data repository in the biomedical field containing high quality datasets for well conducted studies is the BioLINCC repository. The interest to study the characteristics of this data repository is not new, as we pointed in the publications by Coady et al., Ross et al., and Giffen et al. While none of these papers alone provide the full picture of the impact of the BioLINCC repository and open data, they each provide knowledge on certain aspects. We chose to study the number of datasets that were used out of the total datasets in the BioLINCC repository, the number of publications generated (considering that this is the main reason for data requests as found by Ross et al), and number of citations these publications received. While this aim will cover a small aspect of the big question of “the impact of open data”, it will provide an important insight for better understanding of the characteristics of open data at the BioLINCC repository, what are the main datasets used, their fields, and the assurance that using an open dataset won’t compromise publishing potential of studies, if important findings found. We tried to stress further on this point.

**Comment**: The authors have tracked citations to articles that reuse datasets over time. However, with nothing to compare these counts to, it's hard to contextualize what these citations really mean. Are these papers being cited more/less than similar articles that aren't reusing datasets? How do citations to these articles describing secondary reuse compare to the number of citations received by the articles describing the dataset originally and its primary use? It's evident that citations to these articles are going up over time, but that's to be expected to an extent. So I'm not really sure what to make of these numbers and how to use them to understand impact. While this article provides an overview of the state of citations to articles reusing BioLINCC data, it is unclear to me what conclusions can be reasonably drawn from this analysis about impact.

**Response**: We agree with the author that comparing the number of citations received by publications that used open data with primary data articles would provide a better insight into the impact of open data compared to other articles. We amended the aim to narrow its scope so that it accommodates the aspects covered by our study:

 “The impact of open access data, in terms of the number of datasets used from a repository, publications generated from these datasets, and citations received by these publications are still unknown. In this study, we aim to study the BioLINCC datasets, the number of publications that used BioLINCC open access data, and the impact of these publications through the citations they received.”

 We added the point mentioned by the esteemed reviewer to the study limitations and as a suggestion for future studies. We further discussed the aspects regarding the BioLINCC use of datasets and the characteristics of its publications.

# Peer review: Reviewer response for version 1

**DOI:** 10.5256/f1000research.24126.r68865

**Published:** 2020-08-10

**Authors:** Christian Ohmann

**Affiliations:** 1 European Clinical Research Infrastructure Network, ECRIN, Düsseldorf, Nordrhine-Westfalia, Germany

This is an interesting paper about the impact of data sharing for a non-commercial repository (BioLINCC). From the viewpoint of the reviewer, the manuscript should be improved:

 In the section “data collection” the authors describe different data search methods for publications:

1. Updated list of publications received from BioLINCC directly.
2. List of published articles on the BioLINCC website.
3. Manual search of Pubmed with the title of the dataset.

The authors should describe the overlap/differences between the results of the different search strategies, preferably in a figure. The authors could use the PRISMA flow diagram as an example ( https://www.equator-network.org/reporting-guidelines/prisma/).

 The authors state in the “bibliometric analysis” section that “Any study that reported the use of the searched data set as part of its results was included in our analysis”. It is not clear, how the datasets were identified in the publication. Was this performed via the registration number of the underlying study in a registry (e.g. NCT-number) or by the title/acronym of the data set from the BioLINCC database? The authors should clarify how this was performed.

 Important to add would be a statistic describing the number of publications per data set (may be also dependent on the year of publication of the data set in BioLINCC). Are there many datasets without any or only very few publications? Is the majority of publications concentrated in a few datasets? This information is important because no requests for data sharing may not justify costs and resources for preparation of data sharing (e.g. de-identification, curation).

 One of the factors that is relevant for the number of publications is the year when the data set was published in BioLINCC. A figure correlating the date of publication of the data set with the number of publications could illustrate that. This is similar with the relation between the year of publication and the number of citations. These relationships should be worked out in the paper.

 Another aspect to be considered could be the role of outliers in the statistics. Are there datasets and/or publications with a very high number of citations (e.g. more than 100). Does the citation pattern mainly concentrate in a few outstanding datasets or is it more evenly distributed?

 The authors should include and discuss a  cross-sectional web-based survey about access to clinical research data from BioLINCC, covering the period from 2007 to 2014 (Ross JS et al. Data sharing through an NIH central database repository: a cross-sectional survey of BioLINCC users. *BMJ open* 2016;6(9):e012769) ^1^.

 The authors think that it would be good style to thank BioLINCC for providing datasets after contact.

Is the work clearly and accurately presented and does it cite the current literature?

Partly

If applicable, is the statistical analysis and its interpretation appropriate?

Partly

Are all the source data underlying the results available to ensure full reproducibility?

Yes

Is the study design appropriate and is the work technically sound?

Yes

Are the conclusions drawn adequately supported by the results?

Yes

Are sufficient details of methods and analysis provided to allow replication by others?

Partly

Reviewer Expertise:

clinical research, medical informatics

I confirm that I have read this submission and believe that I have an appropriate level of expertise to confirm that it is of an acceptable scientific standard, however I have significant reservations, as outlined above.

## References

[1] : Data sharing through an NIH central database repository: a cross-sectional survey of BioLINCC users.*BMJ Open*.2016;6(9) : 10.1136/bmjopen-2016-012769e01276910.1136/bmjopen-2016-01276927670522PMC5051517

# Response

**Published:** 2020-09-18

**Authors:** Saif Aldeen AlRyalat

**Affiliations:** University of Jordan, Jordan

It is an honor to receive feedback from professor Ohmann, we performed almost all the changes suggested, and we hope the current version satisfies the quality required. Here are the detailed responses.

 This is an interesting paper about the impact of data sharing on a non-commercial repository (BioLINCC). From the viewpoint of the reviewer, the manuscript should be improved:

**Comment**: In the section “data collection” the authors describe different data search methods for publications:

 The updated list of publications received from BioLINCC directly.

 List of published articles on the BioLINCC website.

 Manual search of Pubmed with the title of the dataset.

 The authors should describe the overlap/differences between the results of the different search strategies, preferably in a figure. The authors could use the PRISMA flow diagram as an example ( https://www.equator-network.org/reporting-guidelines/prisma/).

**Response**: Thank you for suggesting the PRISMA flow chart. We added a new flow chart detailing the steps of including datasets and the criteria of inclusion, with the number of datasets resulted after each exclusion step. While the list of publications provided by the BioLINCC was almost complete, the manual and Pubmed searches didn’t yield a significant addition, where only a few articles added only (we added this to the article). On the other hand, the detailed number of datasets included and excluded is of paramount importance, we used the flow chart for detailing its number. 

**Comment**: The authors state in the “bibliometric analysis” section that “Any study that reported the use of the searched data set as part of its results was included in our analysis”. It is not clear, how the datasets were identified in the publication. Was this performed via the registration number of the underlying study in a registry (e.g. NCT-number) or by the title/acronym of the data set from the BioLINCC database? The authors should clarify how this was performed.

**Response**: Any author requested BioLINCC datasets for use in a study should explicitly mention the dataset used in the methods (i.e. the name of the dataset and the acronym if available), in addition to acknowledging the BioLINCC in the acknowledgment section.

**Comment**: Important to add would be a statistic describing the number of publications per data set (may be also dependent on the year of publication of the data set in BioLINCC). Are there many datasets without any or only very few publications? Is the majority of publications concentrated in a few datasets? This information is important because no requests for data sharing may not justify costs and resources for preparation of data sharing (e.g. de-identification, curation).

**Response**: Thank you for this insight. We analyzed the number of publications associated with each dataset. As the reviewer expected, there are many datasets with no publications associated with them, as well as datasets with high number of publications. We added these results and relevant tables, and we further discussed them in the discussion.

**Comment**: One of the factors that is relevant for the number of publications is the year when the data set was published in BioLINCC. A figure correlating the date of publication of the data set with the number of publications could illustrate that. This is similar with the relation between the year of publication and the number of citations. These relationships should be worked out in the paper.

**Response**: The publication year of datasets may vary according to the datasets, and may change with time if the study got updated (i.e. more data released with time). So it was difficult to study it, considering the unavailability of specific dates provided in the dataset we received from the BioLINCC.

**Comment**: Another aspect to be considered could be the role of outliers in the statistics. Are there datasets and/or publications with a very high number of citations (e.g. more than 100). Does the citation pattern mainly concentrate in a few outstanding datasets or is it more evenly distributed?

**Response**: As the author mentioned, we found several “outlier” datasets and we mentioned them in the results. These datasets were associated with higher number of publications compared to other datasets.

**Comment**: The authors should include and discuss a  cross-sectional web-based survey about access to clinical research data from BioLINCC, covering the period from 2007 to 2014 (Ross JS et al. Data sharing through an NIH central database repository: a cross-sectional survey of BioLINCC users. BMJ open2016;6(9):e012769) 1.

**Response**: Thank you for suggesting the article. We made good use of it.

 The authors think that it would be good style to thank BioLINCC for providing datasets after contact.

# Response

**Published:** 2021-01-31

**Authors:** Saif Aldeen AlRyalat

**Affiliations:** University of Jordan, Jordan

Dear Professor Ohmann,

 We hope our responses satisfy your comments, if so, we hope to receive your feedback. 

 Thank you for your time.

 Sincerely,

 Saif Aldeen AlRyalat, MD.

 Corresponding author.
